# Effect of exenatide on heart rate and blood pressure in subjects with type 2 diabetes mellitus: a double-blind, placebo-controlled, randomized pilot study

**DOI:** 10.1186/1475-2840-9-6

**Published:** 2010-01-28

**Authors:** Anne Gill, Byron J Hoogwerf, Jude Burger, Simon Bruce, Leigh MacConell, Ping Yan, Daniel Braun, Joseph Giaconia, James Malone

**Affiliations:** 1 Eli Lilly and Company, Indianapolis, USA; 2 Lilly USA, LLC, Indianapolis, USA; 3 Amylin Pharmaceuticals, Inc, San Diego, USA

## Abstract

**Background:**

Cardiovascular effects of glucose-lowering agents are of increasing interest. Our aim was to assess the effects of the glucagon-like peptide-1 receptor agonist exenatide on heart rate (HR) and blood pressure (BP) in subjects with type 2 diabetes mellitus (T2DM).

**Methods:**

In this double-blind, placebo-controlled trial, subjects with T2DM on metformin and/or a thiazolidinedione were randomized to receive exenatide (5 μg for 4 weeks followed by 10 μg) or placebo BID for 12 weeks. Heart rate and BP were assessed with 24-hour ambulatory BP monitoring. The primary measure was change from baseline in mean 24-hour HR.

**Results:**

Fifty-four subjects (28 exenatide, 26 placebo) were randomized and comprised the intent-to-treat population. Baseline values (exenatide and placebo) were (mean ± SE) 74.4 ± 2.1 and 74.5 ± 1.9 beats/minute for HR, 126.4 ± 3.2 and 119.9 ± 2.8 mm Hg for systolic BP (SBP), and 75.2 ± 2.1 and 70.5 ± 2.0 mm Hg for diastolic BP (DBP). At 12 weeks, no significant change from baseline in 24-hour HR was observed with exenatide or placebo (LS mean ± SE, 2.1 ± 1.4 versus -0.7 ± 1.4 beats/minute, respectively; between treatments, p = 0.16). Exenatide therapy was associated with trends toward lower 24-hour, daytime, and nighttime SBP; changes in DBP were similar between groups. No changes in daytime or nighttime rate pressure product were observed. With exenatide, body weight decreased from baseline by -1.8 ± 0.4 kg (p < 0.0001; treatment difference -1.5 ± 0.6 kg, p < 0.05). The most frequently reported adverse event with exenatide was mild to moderate nausea.

**Conclusions:**

Exenatide demonstrated no clinically meaningful effects on HR over 12 weeks of treatment in subjects with T2DM. The observed trends toward lower SBP with exenatide warrant future investigation.

**Trial registration:**

NCT00516074

## Background

Glucagon-like peptide-1 (GLP-1), an incretin hormone, plays a key role in glucose homeostasis [1]. Wide physiological distribution of the GLP-1 receptor suggests multiple mechanisms of metabolic control, both centrally and through peripheral neurohumoral pathways. Seminal studies in rodent models demonstrate central regulation of GLP-1 receptor activation resulting in dose-dependent increases in heart rate (HR) and blood pressure (BP) [2,3], while other studies suggest peripheral anti-hypertensive effects [4,5]. In higher-animal models and human studies, few chronotropic and hypertensive effects have been observed [6-16]; however, little is understood about the cardiovascular effects of GLP-1 receptor activation in humans.

Exenatide is a GLP-1 receptor agonist approved for the treatment of type 2 diabetes mellitus (T2DM) [17] and has been associated with improvements in systolic BP (SBP) in long-term clinical trials [18-22]. In shorter-term trials, clinical pharmacology studies demonstrated small increases in HR [23,24], which have not been well characterized. In the present study, we assessed effects of exenatide on HR and BP over 12 weeks, with the use of 24-hour BP monitoring, in relatively normotensive subjects with T2DM.

## Methods

### Subjects

Subjects with T2DM moderately well controlled with metformin, a thiazolidinedione, or metformin plus a thiazolidinedione were recruited from 5 centers in Canada and the Netherlands. Entry criteria included 18 to 75 years of age, stable metformin dose for 30 days or thiazolidinedione for 120 days, body mass index >25 and <40 kg/m^2^, hemoglobin A_1c _between 6.5 and 9.5%, and stable body weight (≤ 10% variation for 3 months). Stable antihypertensive regimens were maintained at least 6 weeks.

Subjects were excluded if they had a history of clinically significant cardiac disease or cardiac disease within one year, clinically significant arrhythmia, resting HR <60 or >100 beats/minute, repeated SBP >160 mm Hg or diastolic BP (DBP) >100 mm Hg, or current treatment with beta blockers.

### Study design

In this multicenter, double-blind, placebo-controlled trial, subjects received subcutaneous placebo injections during a 1-week placebo lead-in before randomization to exenatide or placebo BID for 12 weeks. At Week 4, exenatide dose was escalated from 5 μg to 10 μg BID, which was continued for the remaining 8 weeks. Study medication was administered within 60 minutes before morning and evening meals. Metformin and/or thiazolidinedione or antihypertensive medications remained constant.

Subjects were instructed on the use of the ambulatory BP monitor (model 90207; SpaceLabs, Inc.; Redmond, WA). Monitoring was conducted at baseline, before and after first dose, before and after dose escalation (Week 4), and at Weeks 5, 8, and 12. Subjects activated the monitor prior to evening dose (before the evening meal) and deactivated after 25 hours.

In accordance with the Declaration of Helsinki, the institutional review board approved a common clinical protocol for each site. All participants gave informed written consent.

### Measures

The primary measure was change in mean 24-hour HR from baseline to Week 12. Secondary measures included hourly HR and change from baseline in the following: 24-hour, daytime/nighttime HR, SBP, and DBP; rate pressure product (product of HR and SBP); mean arterial pressure (arterial BP of single cardiac cycle); hemoglobin A_1c_; and body weight. "Nighttime" was defined as 12:00 AM to 6:00 AM.

### Statistical analysis

Fifty subjects were to be randomized. Assuming a 24% dropout rate, 19 subjects per treatment would complete the trial, providing 90% power to detect a pre-defined 10-beats/minute treatment difference for the primary measure, assuming a common standard deviation of 9.23 and a two-sided t test at a significance level of 0.05.

The intent-to-treat population was defined as randomized subjects who received at least one dose of study medication. The per-protocol population was defined as intent-to-treat subjects with valid Week-12 ambulatory BP values, no change in antihypertensive medication, and no violation of inclusion/exclusion or discontinuation criteria.

The primary measure was based on the per-protocol population (pre-defined) and assessed by an analysis of covariance model, including treatment and baseline value of the dependent variable as covariate. Least squares (LS) means and p-values were derived from the model to estimate treatment effects. To support the primary analysis, a mixed model repeated measures analysis was performed for the intent-to-treat population.

Secondary HR measures; hemoglobin A_1c_; body weight; and post- hoc analyses of SBP, DBP, rate pressure product, and mean arterial pressure were based on the intent-to-treat population. Secondary HR measures, hemoglobin A_1c_, body weight, rate pressure product, and mean arterial pressure were assessed by an analysis of covariance model similar to the primary analysis. SBP and DBP were assessed by an analysis of variance model.

Adverse events were summarized by frequency and percentage. Hypoglycemia rate was summarized by treatment and visit, with Fisher's exact test for treatment comparisons.

## Results

### Subjects

**Figure 1:**
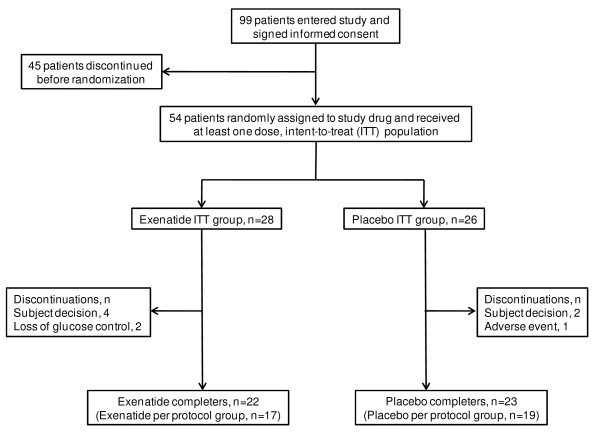
**Patient flow diagram**.

Fifty-four subjects formed the intent-to-treat population, 45 completed the study, and 36 formed the per-protocol population (Figure 1). Table 1 presents baseline characteristics. No statistically significant differences were observed between groups in any of the baseline variables. Patients were mostly overweight and with BP in the early stage-1 hypertensive range.

**Table 1:** baseline subject characteristics

Characteristics at inpatient screening	Exenatide n = 28	Placebo n = 26
Age, years^a^	57 ± 11	54 ± 10

Gender, male, n (%)	19 (68)	11 (42)

Race, n (%)		

Caucasian	24 (86)	25 (96)

African	2 (7)	0 (0)

East Asian	1 (4)	1 (4)

Hispanic	1 (4)	0 (0)

Weight, kg^a^	91.6 ± 15.2	85.9 ± 12.2

BMI, kg/m^2a^	29.5 ± 3.4	30.1 ± 3.9

Hemoglobin A_1c_, %^a^	7.5 ± 0.9	7.1 ± 0.7

Duration of diabetes^a^	7 ± 4	6 ± 4

HR, beats/minute^a^	75.2 ± 12.4	73.7 ± 9.0

SBP, mm Hg^a^	139.4 ± 13.9	138.6 ± 18.7

DBP, mm Hg^a^	83.1 ± 7.4	80.1 ± 12.2

		

Vital signs collected by ambulatory BP monitoring		

HR, beats/minute^a^		

24-hour	74.4 ± 11.0	74.5 ± 9.7

Daytime	75.5 ± 12.0	75.4 ± 10.5

Nighttime	71.2 ± 10.5	71.8 ± 11.1

SBP, mm Hg^a^		

24-hour	126.4 ± 16.6	119.9 ± 14.0

Daytime	128.0 ± 19.4	121.2 ± 14.7

Nighttime	122.0 ± 14.5	116.1 ± 14.8

DBP, mm Hg^a^		

24-hour	75.2 ± 11.1	70.5 ± 9.9

Daytime	77.0 ± 12.4	72.1 ± 10.6

Nighttime	69.8 ± 11.6	65.8 ± 10.4

^a^Mean ± standard deviation; intent-to-treat population shown; BMI, body mass index; BP, blood pressure; DBP, diastolic BP; HR, heart rate; SBP, systolic BP

### Heart rate

No statistically significant change from baseline in 24-hour HR was observed at Week 12 in either group. For exenatide-treated subjects (per-protocol population), LS mean (± SE) 24-hour HR change from baseline was 1.5 ± 1.8 beats/minute (p = 0.41), and for placebo-treated subjects, change from baseline was -0.01 ± 1.71 beats/minute (p = 0.99). There was no treatment difference (p = 0.54). Intent-to-treat results were similar (exenatide, 2.1 ± 1.4 beats/minute, p = 0.13; placebo, -0.7 ± 1.4 beats/minute, p = 0.62; between treatments p = 0.16).

**Figure 2:**
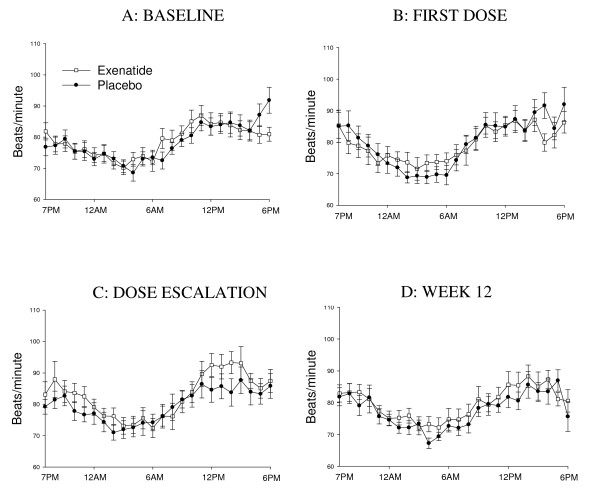
**Hourly heart rates during 24-hour monitoring**. Heart rate values at baseline (A), after first dose (B), after dose escalation (C), and at Week 12 (D) for the intent-to-treat population are shown.

Figure 2 shows hourly HR (intent-to-treat). Overall, exenatide was not associated with significant changes in mean 24-hour, daytime, or nighttime HR within or between groups. A statistically non-significant increase in mean 24-hour HR (approximately 3 beats/minute) relative to placebo was observed with exenatide treatment at weeks 5 and 8, with lesser increases at other times. Of note, immediately after dose escalation, exenatide was associated with a small, transient increase in LS mean 24-hour HR change from baseline (3.4 ± 1.6 beats/minute, p < 0.05). However, nearly half the subjects were missing a valid 24-hour HR value at this time, and results from the mixed models repeated measures analysis (2.3 ± 1.6 beats/minute, p = 0.16) did not support a statistically significant increase.

### SBP, DBP, rate pressure product, and mean arterial pressure

**Figure 3:**
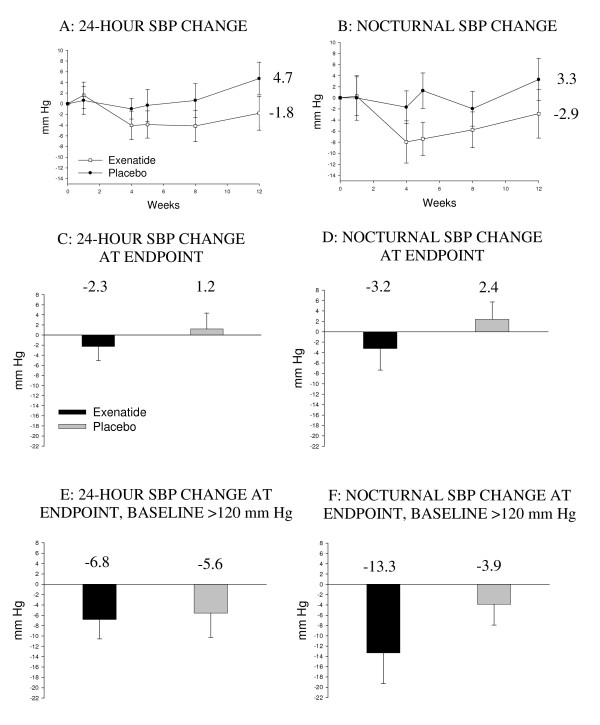
**Effects of exenatide on 24-hour and nocturnal systolic blood pressure (SBP)**. Changes over the study period (A and B), at endpoint (C and D), and for subjects with high (>120 mm Hg) baseline SBP values (E and F) are shown. Seventeen exenatide-treated subjects and 11 placebo-treated subjects had high baseline 24-hour SBP values (E), and 13 exenatide-treated subjects and 9 placebo-treated subjects had high baseline nocturnal SBP values (F). P = NS. Intent-to-treat sample population and mean ± SE are shown. Last observation carried forward for Plots C through F.

Figure 3 shows changes in 24-hour and nocturnal SBP (intent-to-treat). A statistically non-significant trend in decreasing mean 24-hour, daytime, and nighttime SBP (-3.5, -2.9, and -5.6 mm Hg, respectively) was observed with exenatide treatment relative to placebo after 2 weeks of treatment. The trend to lower nocturnal SBP was more prevalent for subjects whose baseline values were >120 mm Hg. No significant changes from baseline in mean 24-hour, daytime/nighttime DBP were observed within or between groups. In post-hoc analyses, no significant changes in daytime/nighttime rate pressure product or mean arterial pressure at Week 12 were observed within or between groups.

### Body weight and hemoglobin A1c

Exenatide reduced mean body weight from baseline by -1.8 ± 0.4 kg (p < 0.0001) at Week 12, whereas placebo resulted in no significant change (-0.3 ± 0.4 kg, p = 0.52). Treatment difference was -1.5 ± 0.6 kg (p < 0.05). There was a -0.3 ± 0.2% reduction in hemoglobin A_1c _for exenatide relative to placebo (p = 0.26).

### Adverse events

Mild or moderate nausea was the most frequently reported adverse event in exenatide-treated subjects (36% of subjects versus 19% for placebo). No episodes of severe hypoglycemia were reported. Incidence of minor hypoglycemia was low and reported in 7% and 4% of subjects who received exenatide and placebo, respectively.

## Discussion

Cardiovascular effects of T2DM medications are of increasing relevance. Exenatide improves glucose homeostasis through several mechanisms, with associated weight loss and low hypoglycemia risk. The aim of the current study was to characterize HR and BP effects of exenatide versus placebo in mostly overweight subjects with BP in the early stage-1 hypertensive range and with T2DM. Exenatide demonstrated no significant effect on HR or BP in this 12-week trial, although trends toward decreased SBP were observed. These observations are consistent with larger, longer-term trials that did not show any apparent cardiovascular safety concerns and suggested some improvement in SBP [18-22].

Glycemic and BP control are additive in improved outcomes in subjects with T2DM. An analysis of the United Kingdom Prospective Diabetes Study demonstrated that any reduction in SBP is likely to reduce the risk of complications in subjects with T2DM [25,26]. A 10-mm Hg (6.5%) reduction in SBP was associated with 11% reduction in any diabetes-related endpoint, including stroke (18%) and myocardial infarction (11%) [25]. In the Hypertension Optimal Treatment study, small decreases in SBP and DBP across treatment groups were associated with reduced major cardiovascular events and cardiovascular mortality in hypertensive subjects with T2DM [27]. Accordingly, modest decreases in SBP, as in the present trial, also may have clinical significance, and further SBP decreases are likely with additional weight loss. Studies of longer duration of exenatide use have suggested improved SBP effects [18-22], notably in subjects with abnormally high baseline SBP values [21]. Integrated analyses of long-term exenatide exposure did not suggest increased risk of exenatide on cardiovascular outcomes [28,29].

These data compare well with previous studies in which GLP-1 receptor activation conferred no effect on HR [11-16] with slight trends toward decreased BP in some cases [11,15]. Exceptions were observed in small cohorts of fasted healthy volunteers [30] and subjects with T2DM [23] in short-term studies, with small, transient increases in HR or BP. In the current study, the trend towards favorable SBP changes is consistent with larger, longer-term trials. No increase in rate pressure product or mean arterial pressure at endpoint in this study also implied no increase in cardiac work load. Data suggesting an increase in HR following dose escalation were not supported by a repeated measures analysis and were considered inconclusive. The observed change in hemoglobin A_1c _is likely related to the relatively low baseline value and short duration of treatment. Multiple published studies demonstrated the glucose-lowering effects of exenatide, typically resulting in 30-60% of subjects achieving hemoglobin A_1c _of ≤ 7.0% when exenatide is added to ongoing therapy [17].

In this trial, the study population of mostly overweight subjects (mean BMI for exenatide, 29.5 ± 3.4 and for placebo, 30.1 ± 3.9 kg/m^2^) with BP in the early stage-1 hypertensive range does not represent all patients with T2DM and is a limitation of the study. The trial was also limited by the sample size which may have been insufficient for determining statistical significance in some non-primary results.

Previous trials with exenatide, some of which included more obese patients and treatment durations over longer periods of time, resulted in greater weight loss and greater reductions in BP than those observed in the present study [17-20]. Though weight reduction improves hypertension, apparent BP trends observed in this study are unlikely solely due to weight loss in view of the treatment period of this duration [31]. Thus, concomitant mechanisms associated with BP trends, in addition to weight loss, seem plausible and are consistent with other findings. In rodents, Golpon et al. [5] proposed direct dilatory effects on peripheral vasculature. GLP-1 dose- and time-dependently relaxed pulmonary arterial rings and reduced pulmonary vascular tone, presumably mediated by nitric oxide and an intact endothelium. However, other studies also suggest the possibility of nitric oxide- and endothelium-independent relaxant effects [32]. In humans, activation of the GLP-1 receptor, also isolated from human coronary artery endothelial cells, resulted in vasodilation of brachial arteries and improved endothelial dysfunction in subjects with T2DM with coronary heart disease [14]. These reports suggest direct activation of the GLP-1 receptor in peripheral endothelial tissue, mediating vasodilatory effects, as a possible mechanism.

The findings of this study suggest that 12-week administration of exenatide had little effect on HR and showed some trends of improved BP effects. Larger subsequent studies should clarify the effect of exenatide on trends in cardiovascular markers, including weight loss and SBP, and explore the potential for lowering overall risk.

## Competing interests

AG, BJH, JB, DB, JG, and JM are employed by and are share holders of Eli Lilly and Company. SB, LM, and PY are employed by and are share holders of Amylin Pharmaceuticals, Inc.

## Authors' contributions

AG, DB, JB, and JM contributed to the design of this study. JB and PY performed the statistical analyses. All authors contributed to the interpretation of the study results and writing process and approved the final manuscript.

## Authors' information

AG is a senior clinical research scientist, BJH and DB are medical fellows, JB is an associate consultant statistician, JG is a medical writer, and JM is senior medical director for diabetes/endocrine, all at Eli Lilly and Company. SB is senior medical director, LM is an associate director of medical research, and PY is a senior statistician at Amylin Pharmaceuticals, Inc.
